# Cardiovascular and inflammatory effects of intratracheally instilled ambient dust from Augsburg, Germany, in spontaneously hypertensive rats (SHRs)

**DOI:** 10.1186/1743-8977-7-27

**Published:** 2010-09-29

**Authors:** Swapna Upadhyay, Koustav Ganguly, Tobias Stoeger, Manuela Semmler-Bhenke, Shinji Takenaka, Wolfgang G Kreyling, Mike Pitz, Peter Reitmeir, Annette Peters, Oliver Eickelberg, H Erich Wichmann, Holger Schulz

**Affiliations:** 1 Comprehensive Pneumology Center, Institute of Lung Biology and Disease, Helmholtz Zentrum München, German Research Center for Environmental Health, Ingolstaedter Landstrasse 1, D85764, Neuherberg/Munich, Germany; 2 Focus Network Nanoparticles and Health, German Research Center for Environmental Health, Neuherberg, Germany. Ingolstaedter Landstrasse 1, D85764, Neuherberg/Munich, Germany; 3 Institute of Epidemiology, Helmholtz Zentrum München, German Research Center for Environmental Health, Ingolstaedter Landstrasse 1, D85764, Neuherberg/Munich, Germany; 4 Institute of Health Economics and Health Care Management, Helmholtz Zentrum München, German Research Center for Environmental Health, Neuherberg, Germany. Ingolstaedter Landstrasse 1, D85764, Neuherberg/Munich, Germany

## Abstract

**Rationale:**

Several epidemiological studies associated exposure to increased levels of particulate matter in Augsburg, Germany with cardiovascular mortality and morbidity. To elucidate the mechanisms of cardiovascular impairments we investigated the cardiopulmonary responses in spontaneously hypertensive rats (SHR), a model for human cardiovascular diseases, following intratracheal instillation of dust samples from Augsburg.

**Methods:**

250 μg, 500 μg and 1000 μg of fine ambient particles (aerodynamic diameter <2.5 μm, PM_2.5_-AB) collected from an urban background site in Augsburg during September and October 2006 (PM_2.5 _18.2 μg/m^3^, 10,802 particles/cm^3^) were instilled in 12 months old SHRs to assess the inflammatory response in bronchoalveolar lavage fluid (BALF), blood, lung and heart tissues 1 and 3 days post instillation. Radio-telemetric analysis was performed to investigate the cardiovascular responses following instillation of particles at the highest dosage based on the inflammatory response observed.

**Results:**

Exposure to 1000 μg of PM_2.5_-AB was associated with a delayed increase in delta mean blood pressure (ΔmBP) during 2^nd^-4^th ^day after instillation (10.0 ± 4.0 vs. -3.9 ± 2.6 mmHg) and reduced heart rate (HR) on the 3^rd ^day post instillation (325.1 ± 8.8 vs. 348.9 ± 12.5 bpm). BALF cell differential and inflammatory markers (osteopontin, interleukin-6, C-reactive protein, and macrophage inflammatory protein-2) from pulmonary and systemic level were significantly induced, mostly in a dose-dependent way. Protein analysis of various markers indicate that PM_2.5_-AB instillation results in an activation of endothelin system (endothelin1), renin-angiotensin system (angiotensin converting enzyme) and also coagulation system (tissue factor, plasminogen activator inhibitor-1) in pulmonary and cardiac tissues during the same time period when alternation in ΔmBP and HR have been detected.

**Conclusions:**

Our data suggests that high concentrations of PM_2.5_-AB exposure triggers low grade PM mediated inflammatory effects in the lungs but disturbs vascular homeostasis in pulmonary tissues and on a systemic level by affecting the renin angiotensin system, the endothelin system and the coagulation cascade. These findings are indicative for promotion of endothelial dysfunction, atherosclerotic lesions, and thrombogeneis and, thus, provide plausible evidence that susceptible-predisposed individuals may develop acute cardiac events like myocardial infarction when repeatedly exposed to high pollution episodes as observed in epidemiological studies in Augsburg, Germany.

## Introduction

Epidemiological studies have linked ambient particulate matter (PM) levels to an increased incidence of adverse cardiovascular events. The World Health Organization [1] reported that over 800,000 premature deaths worldwide per year can be attributed to PM air pollution [1]. Numerous epidemiological studies have linked short-term and long-term PM exposures to increased mortality [2-5]. An analysis by Pope et al. [6] indicated that with every 10 μg/m^3 ^increase in atmospheric PM_2.5_, cardiopulmonary mortality increased by 6%. For a similar increase in PM_2.5_, it has been shown that there is a 2.1% increase in the number of deaths related to ischemic heart disease [7]. Short-term increases in PM exposure elevate the incidence of ischemic cardiac disease and congestive cardiac failure with specific pathophysiological end points like myocardial infarction, arrhythmias, reduced heart-rate variability (HRV), and elevated heart rate (HR) in adults [5,8-12]. Inhalation of PM may cause pulmonary inflammation characterized by neutrophil and macrophage activation [13-16] associated with a subsequent systemic inflammatory response and the disturbance of endothelial function and activation of the blood coagulability [15-19]. Repeatedly, PM was associated with an increase in serum levels of C-reactive protein, the classical acute phase reactant indicative for a systemic inflammatory response which is considered to be a risk factor for cardiovascular diseases [7,15,20,21]. However, the underlying pathophysiological mechanisms of airborne PM_2.5 _mediated cardiopulmonary mortality and morbidity are complex and remain to a large extent unexplored.

**Figure 1:**
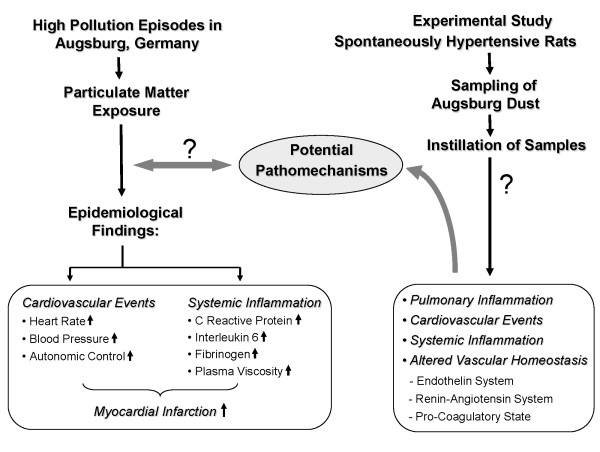
**The figure illustrates the rationale of experimental study evolving from the epidemiological findings**.

Several epidemiological studies on PM associated cardiovascular effects were conducted in Augsburg, a medium sized city located in Southern Germany with about 250,000 inhabitants and no heavy industry, by using the 'Cooperative Health Research in the Region of Augsburg (KORA)' platform. In January 1985, an air pollution episode occurred throughout Central Europe [22] resulting in an increase of hospital admissions for cardiovascular diseases, such as acute coronary syndromes and arrhythmias [23]. The first MONICA survey (MONI*toring *of trends and determinants in Cardiovascular disease) was carried out in Augsburg during that winter period which included the days of the pollution episode [24]. Peters et al [20] reported that PM in the Augsburg atmosphere may have induced systemic inflammation as suggested by increased C-reactive protein levels and increased plasma viscosity [17]. In several epidemiological studies, exposure to Augsburg dust was associated with an increase in HR [9], arterial BP and an altered autonomic control [25] as well as the increased incidences of myocardial infarction among susceptible persons [[5,26]; Figure 1). Since 1985 exposure to airborne PM has changed in Augsburg mainly due to modified emissions of combustion sources. It continues to pose threat to the population in Augsburg for various alignments mainly cardiopulmonary disorders.

Therefore investigations on the causative pathomechanism for cardiovascular diseases due to PM exposure will help immensely in understanding the molecular phenomenon and support preventive, such as creation of low emission zones, or curative strategies. To address the potential underlying pathomechanism for PM associated cardiovascular diseases, spontaneously hypertensive rats (SHRs; 12 months), a well established animal model of human cardiovascular disease were exposed to PM_2.5_-AB collected during September and October 2006 by intratracheal (i.t.) instillation and assessed for cardiopulmonary responses, its temporal assignment, and its potential molecular basis. We hypothesized that either by direct particle-cell interaction or by inflammatory responses in the lungs and/or on the systemic level exposure to PM_2.5_-AB induces markers of endothelial dysfunction which promotes a pro-coagulatory state and may induce areteriosclerotic lesions. Exposure also affects the vasoregulatory homeostasis by affecting the endothelin and the renin-angiotensin system (RAS) resulting in an increased arterial BP which ultimately contribute to an increased afterload and oxygen demand of the heart. We expect effects of a single exposure to be small and, taken as a unique event, to be clinically of minor importance in subjects with sufficient cardiovascular reserves. However, repeated exposures over several years as given in the environmental setting of Augsburg is supposed to promote cardiovascular disorders and eventually contribute to trigger fatal cardiac events in susceptible subjects such as myocardial infarction documented in the epidemiological studies. In the present study we focused on the acute response detectable within 5 days after a single instillation of PM_2.5_-AB and analysed a panel of protein markers assessing vasoregulatory pathways (Endothelin-1, RAS) and markers indicative for coagulation and atherosclerosis (PAI-1;TF; VCAM-1) and angiogenesis (VEGF). Our findings provide evidence for plausible molecular mechanisms underlying low grade PM_2.5_-AB mediated inflammatory effects and alterations of the vascular homeostasis. We could show that a single exposure of SHRs to PM_2.5_-AB induces a slight inflammatory response in pulmonary and systemic tissues, including activation of the endothelial and vasoregulatory system and promotion of a pro-coagulatory state. This may contribute to the development of cardiovascular disorders and to cardiac events in susceptible persons as observed in epidemiological studies in Augsburg (Figure 1).

**Figure 2:**
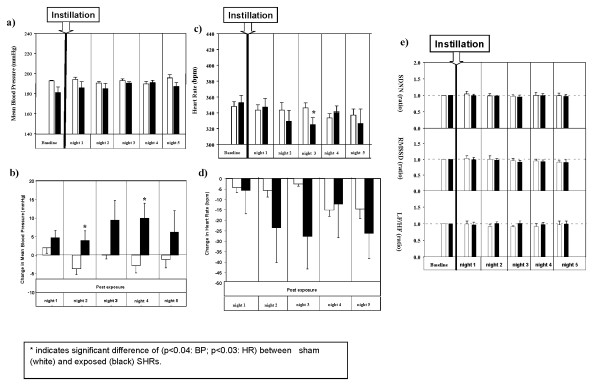
**Mean blood pressure (mBP, 2a); Changes in individual mean blood pressure (delta mBP, 2b).; Heart rate (HR, 2c); Changes in individual heart rate (delta HR, 2d); and Changes in time domain and frequency domain measures of heart rate variability (HRV, 2e) in sham (control) or Augsburg particulate matter (PM_2.5_-AB, exposed) instilled SHRs**. The vertical bars exhibit arithmetic mean values (mean ± SE) of sham (white; n = 5) and exposed (black; n = 5) groups. The summary statistics for the analysis of repeated measurements revealed that delta mBP differences between groups were significant on day 2 and 4 post exposure (p < 0.04). Delta mBP declined and reached almost baseline values on day 5.. Absolute HR values responded with a lag of 1 day to PM_2.5_-AB exposure and were significantly lower in the exposure group by 6% (325.1 ± 8.8 bpm vs 348.9 ± 12.5 bpm, p < 0.03) on the 3^rd ^day after instillation. The summary statistics for the analysis of repeated measurements revealed that neither the area under the curve nor differences in HR values reached the level of statistical significance. In Figure 2e each bar represents a combined mean value of: 12 5-minutes segments/12-h dark period/rat) from 5 rats. SDNN: standard deviation of normal to normal (NN) intervals. RMSSD: square root of the mean of squared differences between adjacent NN intervals. LF/HF: ratio of the absolute powers in the low-frequency (LF: 0.20 Hz to 0.75 Hz) and high-frequency bands HF: 0.75 Hz to 2.5 Hz). * indicates significant difference of (p < 0.04: BP; p < 0.03: HR) between exposed (black) and sham (white) SHRs.

## Results

SHR were intra tracheally (i.t.) instilled with ambient PM_2.5 _collected from Augsburg, Germany, and were assessed for cardiophysiological response using radio telemetry on 5 consecutive days post exposure. To assess inflammatory responses in the lung and on a systemic level standard bronchoalveolar lavage fluid (BALF), blood samples, and tissue samples from heart and lung were obtained on day 1 and 3 after instillation. Histopathological studies were carried out in lung and heart. Protein concentrations of various inflammatory markers were measured in BALF, lung and heart. Focused analysis of biomarkers (at protein level) associated with hypertension (angiotensin converting enzyme: ACE), endothelial activation (endothelin-1: ET-1), coagulation factors (tissue factor: TF; plasminogen activator inhibitor-1: PAI-1), atherosclerosis (vascular cell adhesion molecule 1: VACM-1) and angiogenesis (vascular endothelial growth factor: VEGF) was performed from the lung and heart. Sham (water/vehicle) instilled animals served as control.

### Cardiovascular response assessed by radio telemetry

To address the PM_2.5_-AB induced cardiovascular response a well established animal model (spontaneously hypertensive rats, SHRs) of human cardiovascular disease has been used in the present study. All animals (SHRs; 12 months) showed clear signs of cardiovascular disease at the time of investigation. Mean blood pressure (mBP) of unexposed rats ranged between 169 and 208 mmHg and mean heart weight between 1463 and 2112 mg, thus, being substantially elevated compared to non-hypertensive WKY rats [27,28]. Despite being kept under identical housing conditions the extent of hypertension and its effects on secondary organs varied between animals. The animals were randomly assigned to the control or exposure group, respectively.

Figure 2 summarizes effects on the cardiovascular system following instillation of 1000 μg of PM_2.5_-AB. After PM_2.5_-AB exposure no significant differences between control and exposed animals were detectable for absolute values of mBP (Figure 2a). However, the interpretation of mBP data based on absolute values is limited in this case because baseline data were 9% lower in exposed than in control animals (exposed: 181.1 ± 4.8 mmHg versus control: 197.0 ± 2.8 mmHg, p < 0.02). Differences in mBP were due to a moderate changes in systolic (exposure: 199.6 ± 4.0 mmHg versus control: 226 ± 2.5 mmHg) and diastolic (exposure: 162.5 ± 6.1 mmHg versus control/168 ± 3.1 mmHg) BP. Therefore, individual mBP differences (ΔmBP) were calculated from post exposure values related to the respective baseline (Figure 2b). The summary statistics for the analysis of repeated measurements (AUC statistics) revealed that differences in control animals varied randomly between -3.8 ± 1.5 mmHg (day 2 post exposure) and +2.0 ± 1.6 mmHg (day 1 post exposure) while those observed in exposed animals increased with a lag of 1 day and peaked on post exposure day 4 (+10.0 ± 4.0 mmHg, p < 0.05). Delta mBP differences between groups were significant on day 2 and 4 post exposure (p < 0.04, paired t-test). Equivalent changes were observed on day 3, although statistically not significant (p < 0.1, paired t-test). Delta mBP declined and reached almost baseline values on post exposure day 5 (6.2 ± 5.8 mmHg; not significant).

Baseline measurements of HR revealed comparable values between both groups, i.e. 351.6 ± 5.7 and 352.8 ± 9.4 in control and exposed animals, respectively (Figure 2c, not significant). Absolute HR values responded with a lag of 1 day to PM_2.5_-AB exposure and were significantly lower in the exposure group by 6% (325.1 ± 8.8 bpm vs 348.9 ± 12.5 bpm, p < 0.03) on the 3^rd ^post exposure day. However, applying the same statistical approaches as used for mBP neither the area under the curve nor differences in HR values (Figure 2d) reached the level of statistical significance.

The standard deviation of all normal adjacent sinus intervals (SDNN), a measure of the overall heart-rate variability (HRV), the square root of the mean of squared differences between adjacent normal to normal intervals (RMSSD) and the low-frequency to high-frequency ratio (LF/HF) remained unaffected in exposed SHRs compared to their control (Figure 2e).

### Acute pulmonary inflammatory response

**Figure 3:**
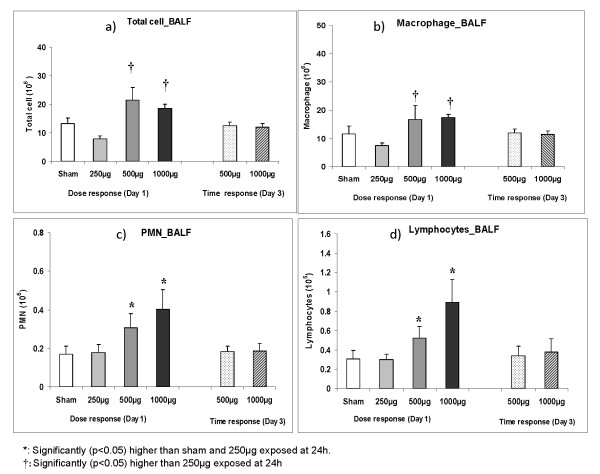
**Total lavagable cells (2a), absolute macrophage (2b), polymorphonuclear leukocytes (PMN; 2c) and lymphocytes (2d) numbers of brcheoalveolar lavage fluid (BALF) obtained from sham (control) and Augsburg particulate matter (PM_2.5_-AB, exposed) instilled SHRs**. Dose (250 μg to 1000 μg) and time dependent changes on day 1 and 3 (24 h and 72 h after instillation) are displayed. Bars represent arithmetic mean values ± SE of sham (n = 6) and exposure groups (n = 6). **†: **Significantly (p < 0.05) higher than 250 μg exposed at 24 h *: Significantly (p < 0.05) higher than sham and 250 μg exposed at 24 h.

BALF and lung: Typically for SHRs, elevated cell numbers were detected compared to healthy rats [29]. Intratracheal instillation of PM_2.5_-AB resulted in a slight increase of BALF derived inflammatory cells at day 1 post instillation (Figure 3a). Dose dependent increases in macrophages (Figure 3b), polymorphonuclear leukocytes (PMNs) (Figure 3c), and lymphocytes (Figure 3d) occurred in on day 1 (24 h) in 500 μg and 1000 μg PM_2.5_-AB exposed animals. The total lavagable cells, especially the PM_2.5_-AB associated influx of PMN and lymphocytes were largely reversed by day 3 (72 h) and reached baseline values. At the exposure dose of 250 μg total lavageable cells and cell differentials did not exhibit any significant difference compared to sham.

**Figure 4:**
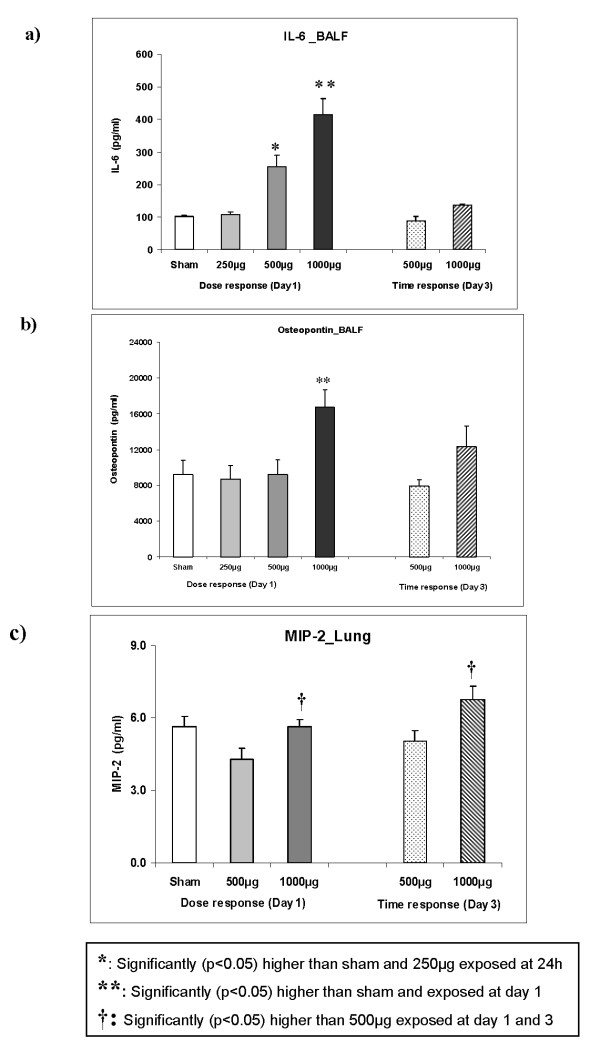
**Response of pulmonary inflammation markers in the bronchoalveolar lavage fluid (BALF)**. Concentrations of Interleukin-6 (IL-6) (3a), osteopontin (3b), and macrophage inflammatory protein-2 (MIP-2) in lung homogenate (3c) following intratracheal (i.t.) instillation of sham and Augsburg particulate matter (PM_2.5_-AB, exposed) in SHRs are shown. Bars represent arithmetic mean values ± SE of sham (n = 6) and exposure groups (n = 6). IL-6 exhibited a typical dose-time response in the BALF. Osteopontin in the BALF and MIP-2 in the lung homogenate (3c) was affected only at the highest dose (1000 μg). *: Significantly (p < 0.05) higher than sham and 250 μg exposed at 24 h **: Significantly (p < 0.05) higher than sham and exposed at day 1. **†: **Significantly (p < 0.05) higher than 500 μg exposed at day 1 and 3

To characterize the degree of inflammatory changes in the lung at a molecular level, we analyzed key inflammatory markers [30] at protein level (IL-6 and osteopontin; Figures 4a-b) from BALF and lung homogenate (macrophage inflammatory protein-2/MIP-2 and tumor necrosis factor alpha/TNF-α; Figure 4c). IL-6 was induced by 2.5 fold and 4.0 fold (p < 0.05; Figure 4a) in the BALF on day 1 post instillation of 500 μg and 1000 μg PM_2.5_-AB, respectively, compared to sham. Osteopontin concentration in BALF was significantly increased by 2.0 fold only at the highest dose (1000 μg; p < 0.05; Figure 4b) compared to sham at day 1 post instillation. Both IL-6 and osteopontin concentrations were reversed by day 3 and reached near to baseline values.

Concentrations of MIP2 in the lung were only slightly affected (Figure 4c). Intratracheal instillation of 1000 μg PM_2.5_-AB significantly increased the MIP-2 (1.3 fold; p < 0.05) concentration at the day 1 and 3 post instillation compared to their corresponding 500 μg exposed group but was not significantly affected compared to sham. TNF-α expression was not significantly altered in any of the doses (data not shown).

#### Pulmonary histopathology

**Figure 5:**
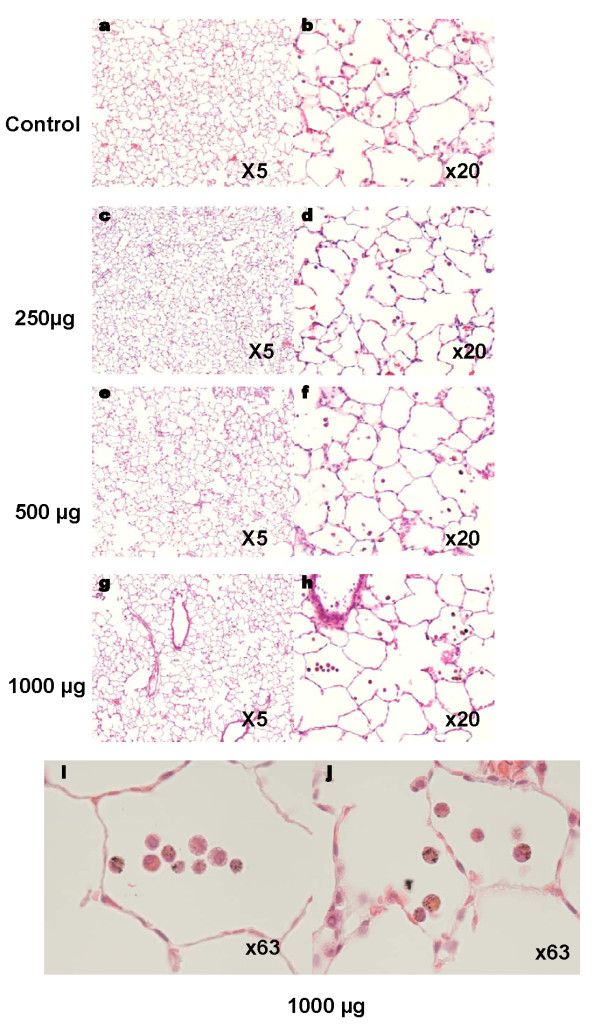
**Lung histology from control and exposed rats**. Light microscopy examination of representative lung sections from sham (a-b) and exposed rats 24 hours after 250 μg (c-d), 500 μg (e-f), or 1000 μg (g-j) PM_2.5_-AB instillation, respectively. Light microscopy examination at higher magnification revealed PM-laden macrophages after instillation of 1000 μg PM_2.5 _Figures 3i and j show two different microscopic fields.

Pulmonary histopathological analysis by light microscopy examination of representative lung sections revealed moderate accumulation of mononuclear cells in the lungs due to the advanced cardiovascular state of the animals [29,31]. A slight inflammatory response with some PMN influx and particle-laden macrophages, were detectable at high doses of PM_2.5_-AB (Figure 5i and 5j). There were no signs of PM_2.5_-AB induced lung injury, even at the highest dose applied.

#### Systemic inflammatory response

##### Acute phase response

**Figure 6:**
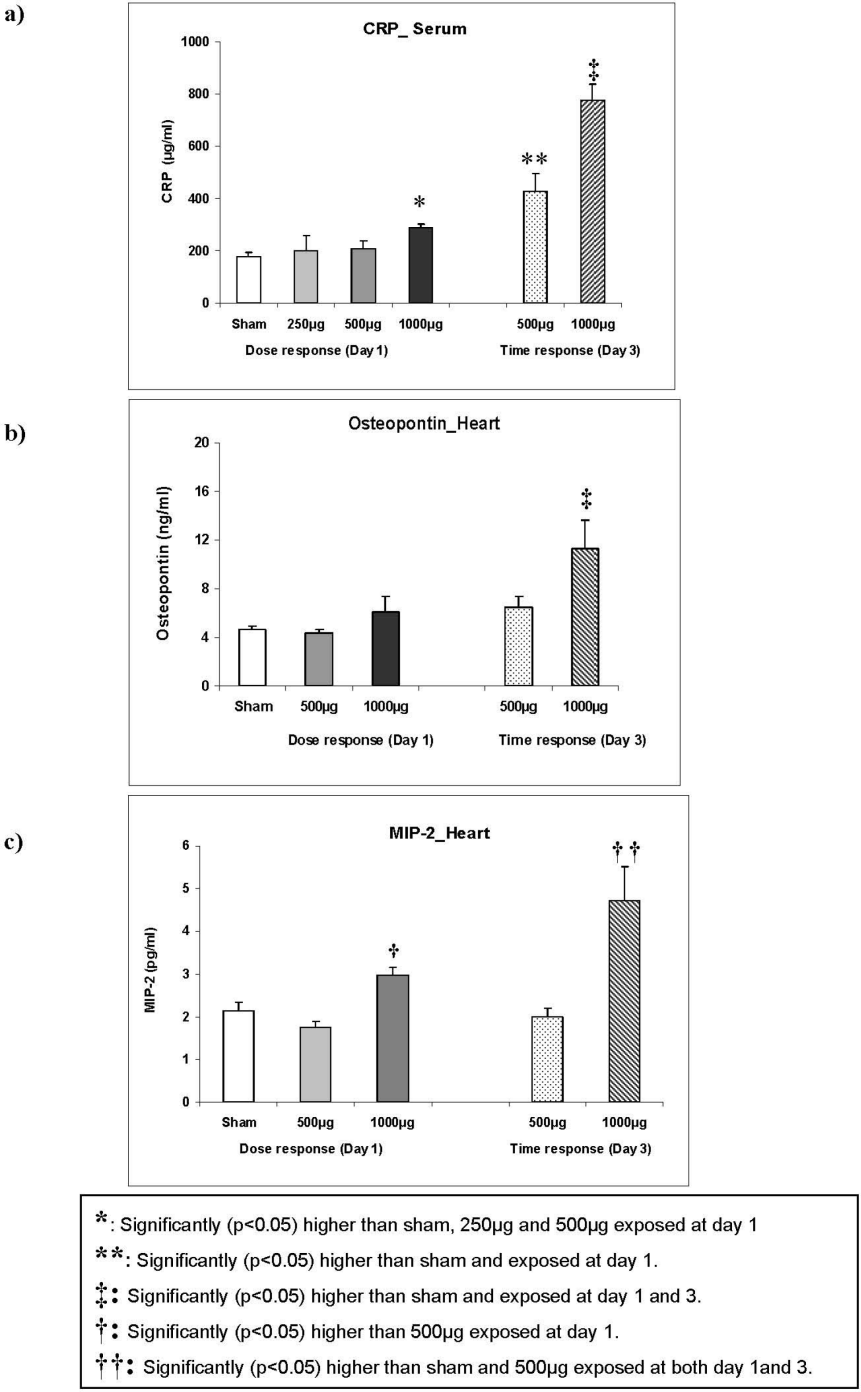
**Response of the systemic Inflammatory marker C-reactive protein (CRP) in the serum (3a), and of osteopontin (3b) and macrophage inflammatory protein 2/MIP-2 (3c) in the heart following intratracheal instillation of sham and Augsburg particulate matter (PM_2.5_-AB, exposed) in spontaneously hypertensive rats (SHRs)**. Bars represent arithmetic mean values ± SE of sham (n = 6) and exposure groups (n = 6). A delayed increase of CRP was observed in a dose-dependent manner whereas osteopontin and MIP2 were induced only at the highest dose. *: Significantly (p < 0.05) higher than sham, 250 μg and 500 μg exposed at day 1. **: Significantly (p < 0.05) higher than sham and exposed at day 1. **‡: **Significantly (p < 0.05) higher than sham and exposed at day 1 and 3. **†: **Significantly (p < 0.05) higher than 500 μg exposed at day 1. **††: **Significantly (p < 0.05) higher than sham and 500 μg exposed at both day 1and 3.

To assess whether PM_2.5_-AB instillation induces an inflammatory response at the systemic level, serum levels of the "acute phase reactant", C-reactive protein (CRP) was determined. A clearly delayed increase of CRP was observed in a dose-dependent manner (Figure 6a) with a 4.4 fold induction of CRP on day 3 following instillation of 1000 μg PM_2.5_-AB (p < 0.05) and a 2.4 fold induction at 500 μg (p < 0.05). At day 1 CRP was not affected after 500 μg PM_2.5_-AB albeit slightly but significantly (1.6 fold; p < 0.05) increased after instillation of 1000 μg compared to sham.

##### Haematology

The complete blood cell count, i.e. total red (haematocrit) and white blood cells, (platelets, neutrophils and lymphocytes) was assessed on 1^st ^and 3^rd ^day after instillation and revealed no alterations between sham and exposed animals.

##### Cardiac tissue

A delayed gradual induction of osteopontin and MIP-2 was observed in cardiac tissue (Figures 6b and 6c). Osteopontin was significantly induced by 2.4 fold only at the highest dose (1000 μg; Figure 6b; p < 0.05) on day 3 post instillation. MIP-2 reflected a similar response as it is observed in the pulmonary tissue. At 1000 μg PM_2.5_-AB MIP-2 was significantly induced by 1.5 fold at day 1 and by 2 fold (p < 0.05; Figure 6c) at day 3 compared to sham. TNF-α was not affected (data not shown).

### PM_2.5 _mediated effects on vascular homeostasis

#### Pulmonary protein analysis

**Figure 7:**
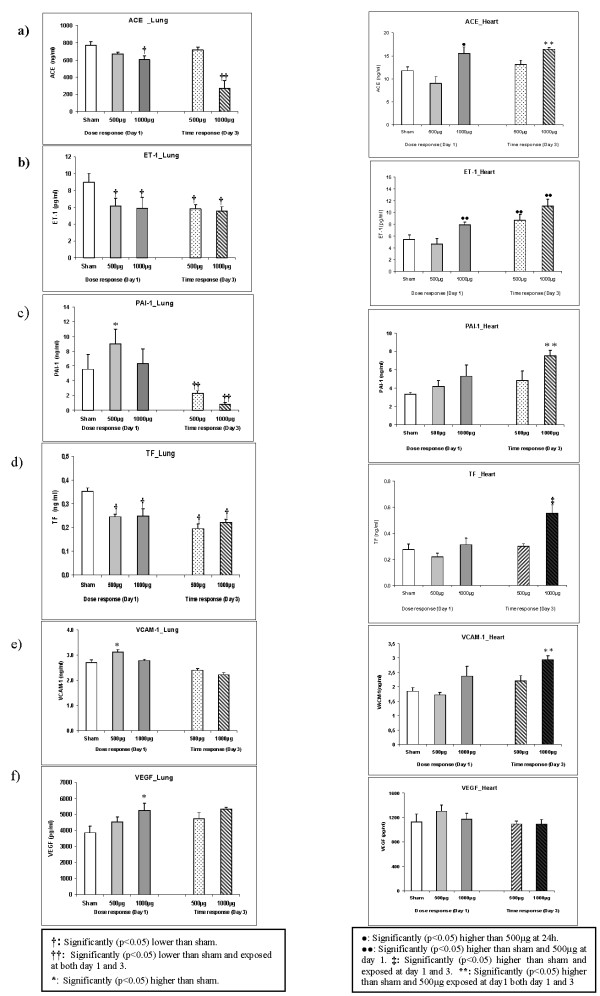
**Side by side comparison of the lung and heart response of angiotensin converting enzyme (ACE; 5a), Endothelin-1 (ET-1; 5b), plasminogen activator inhibitor-1 (PAI-1; 5c), tissue factor (TF; 5d), vascular cell adhesion molecule-1(VCAM-1; 5e) and vascular endothelial growth factor (VEGF; 5f) following intratracheal instillation of sham and Augsburg particulate matter (PM_2.5_-AB, exposed) in spontaneously hypertensive rats (SHRs)**. Bars represent arithmetic mean values ± SE of sham (n = 6) and exposure groups (n = 6). **†: **Significantly (p < 0.05) lower than sham. **††: **Significantly (p < 0.05) lower than sham and exposed at both day 1 and 3. *: Significantly (p < 0.05) higher than sham. **: Significantly (p < 0.05) higher than sham and 500 μg exposed at day1 both day 1 and 3. •: Significantly (p < 0.05) higher than 500 μg at 24 h. ••: Significantly (p < 0.05) higher than sham and 500 μg at day 1. **‡: **Significantly (p < 0.05) higher than sham and exposed at day 1 and 3.

ACE, ET-1, TF, PAI-1, VACM-1 and VEGF protein concentrations were assessed from lung tissues of PM_2.5_-AB exposed SHRs on day 1 and day 3 post instillation (Figures 7a-f; see Table 1). ET1, and TF were significantly reduced by about 35% (1.5 fold repression, p < 0.05) at both PM_2.5_-AB concentrations and did not recover over the time period of investigation. ACE was affected only at the highest concentration (1000 μg). ACE levels decreased to about 75% of its activity (1.3 fold repression, p < 0.05) at day 1 and exhibited an even stronger decrease to about 35% (3 fold repression, p < 0.05) on day 3. PAI-1 (1.5 fold) and VCAM-1 (1.3 fold) were significantly (p < 0.05) increased following instillation of 500 μg of PM_2.5_-AB on day 1, while VEGF was significantly induced (1.5 fold; p < 0.05) only at the highest dose (1000 μg) compared to sham. For PAI-1 an initial increase was not detected at the highest concentration but on day 3 a strong reduction at both 500 and 1000 μg of PM_2.5_-AB to 40% and 15%, respectively (2.4 and 6.5 fold repression, respectively, p < 0.05) was observed.

**Table 1:** Representation of the response pattern (dose and time) of all the markers analyzed in lung and heart tissue following instillation of 500 μg and 1000 μg Augusburg dust in Spontaneously hypertensive rats (SHRs).

Protein	Lung				Heart			
	
	Day1		Day3		Day1		Day3	
	
	500 μg	1000 μg	500 μg	1000 μg	500 μg	1000 μg	500 μg	1000 μg
Interleukin 6 (IL-6)	2.5 fold↑	4.0 fold↑	n.s	n.s	n.d	n.d	n.d	n.d

Osteopontin (SPP1)	n.s	2.0 fold↑	n.s	n.s	n.s	n.s	n.s	2.4 fold↑

Macrophage inflammatory protein 2 (MIP2)	n.s	1.3 fold↑	n.s	1.3 fold↑	n.s	1.5 fold↑	n.s	2.0 fold↑

Tumor necrosis factor alpha (TNFα	n.s	n.s	n.s	n.s-	n.s	n.s	n.s	n.s

Angiotensin converting enzyme (ACE)	n.s	1.3 fold ↓	n.s	3.0 fold ↓	n.s	1.4 fold ↑	n.s	1.4 fold ↑

Endothelin 1 (ET1)	1.5 fold↓	1.5 fold ↓	1.5 fold ↓	1.5 fold ↓	n.s	1.5 fold ↑	1.5 fold ↑	2.1 fold ↑

Plasminogen activator inhibitor 1 (PAI-1)	1.5 fold ↑	n.s	2.4 fold↓	6.5 fold ↓	n.s	n.s	n.s	2.0 fold ↑

Tissue factor (TF)	1.5 fold↓	1.5 fold ↓	1.5 fold ↓	1.5 fold ↓	n.s	n.s	n.s	2.0 fold ↑

Vascular cell adhesion molecule 1 (VCAM1)	1.3 fold↑	n.s	n.s	n.s	n.s	n.s	n.s	1.6fold ↑

Vascular endothelial growth factor (VEGF)	n.s	1.5 fold ↑	n.s	n.s	n.s	n.s	n.s	n.s

n.d: not detected; n.s: not significant.

#### Cardiac protein analysis

The markers assessed in lung were also measured in the heart tissue at day 1 and 3 after instillation (Figures 7a-f; see Table 1). ACE was induced by 1.4 fold at 1000 μg (p < 0.05) on day 1 and 3 post instillation compared to sham. Similarly, ET-1 was significantly induced by 1.5 fold (p < 0.05) on day 1 and 3 following instillation of 1000 and 500 μg of PM_2.5 _compared to sham. At 1000 μg of PM_2.5_-AB on day 3 post instillation ET-1 was induced by 2.1 fold (p < 0.05). On day 3 post instillation almost 2 fold (p < 0.05) elevated concentrations of PAI-1 and TF were detected. VCAM-1 was induced by 1.6 (p < 0.05) fold at 1000 μg of PM_2.5_-AB compared to sham. VEGF was not affected in the heart tissue. As evident, in contrast to the pulmonary response most of these markers exhibit a delayed induction in the cardiac tissue with time (day 3 post instillation) matching the cardiophysiological alterations.

### Cardiac histopathology

No signs of inflammation or cardiomyopathy following instillation of ambient particle could be detected by histological analysis (data not shown).

## Discussion

**Figure 8:**
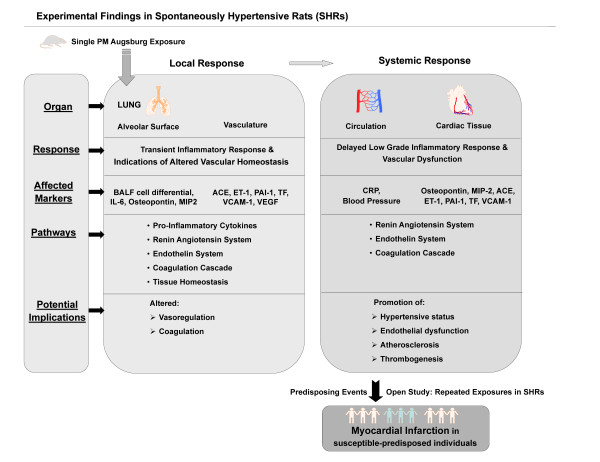
**A schematic summarization of the results obtained from Augsburg dust exposures in spontaneously hypertensive rats (SHRs) in different organs are presented**. **BALF: **Bronchoalveolar lavage fluid; **IL-6: **Interleukin-6; **MIP-2: **Macrophage inflammatory protein-2; **ACE: **Angiotensin converting enzyme**; ET-1: **Endothelin-1; **PAI-1: **Plasminogen activator inhibitor-1; **TF: **Tissue factor; **VCAM-1: **Vascular cell adhesion molecule-1 and **VEGF: **Vascular endothelial growth factor.

Epidemiological studies have identified exposure to elevated concentrations of ambient PM as a risk factor for the exacerbation of ischemic heart disease and congestive heart failure [32,33]. Epidemiological findings in the region of Augsburg, Germany, showed that elevated levels of PM are associated with an increased incidence of myocardial infarction [5,26] and also with increased levels of markers indicative for early systemic effects, like CRP and IL-6, or with plasma viscosity [9,17,20,26,34]. Therefore, it becomes apparent that air pollution events in Augsburg influence cardiopulmonary physiology. Hence, the authors propose that a PM induced low grade, sustained systemic inflammation and modulation of the autonomic function of the heart and the vasomotor activity are potential underlying mechanisms for fatal cardiac event as also suggested by several other epidemiological studies [5,9,10,17,25,26]. As the selections of epidemiological cohorts in most cases are random, the findings are more generalized. However, the case-control study in survivors of myocardial infarction, by Peters et al. (26,10) has reported that transient exposure to traffic may increase the risk of myocardial infarction.. Myocardial infarction is an acute event predisposed due to chronic physiological impairment, like atherosclerotic lesions, a pro-coagulatory state and increased plasma viscosity. Therefore, in this work the experimental design was to study PM_2.5_-AB induced pathways potentially associated with the preceding cardiovascular events that eventually contribute to the observed fatal outcomes reported by epidemiology (Figure 8). In a first approach we focused on the acute response after a single instillation of moderate to high PM_2.5_-AB concentrations in the range of doses applied in other studies [35,36] supposed to cover the range of "no effects" to "obvious response" but being beyond the level of lung injury as shown by biological markers and histological examinations. To identify PM_2.5_-AB induced pathways we investigated two issues in a well established animal model (spontaneously hypertensive rats) of human cardiovascular disease: a) the dose-time dependent inflammatory response of PM_2.5_-AB in the lungs, on a systemic level and the target organ heart complemented by analysis of the cardiovascular performance by radiotelemetry and b) potential pathophysiological mechanisms affecting the cardiopulmonary system following PM_2.5_-AB exposure (Figure 8). We analysed all biomarkers at the protein level compared to mostly transcript based assessments in other studies.

High volume filter samples of ambient Augsburg dust were taken during September and October 2006 at an urban background site in Augsburg. This location has been carefully selected by comparing PM burdens at different urban background sites to find out a representative measurement site for Augsburg [37]. Moreover, since 2004 different online measurement methods were established at this site to characterize the physical and chemical properties of ambient Augsburg dust [38-43]. The main PM sources in urban areas are of anthropogenic origin and therefore a mixture of different particulate emissions like traffic, domestic heating and small-scale industry will be expected. Chemical analysis of PM from Augsburg urban area reported the presence of anthropogenic polycyclic aromatic hydrocarbons (PAH) and their oxidised derivatives (O-PAH).The online measurements during the sample period suggests a moderate PM exposure. For example, the PM_2.5 _mass concentration was 19.8 and 18.2 μg/m^3 ^for the mean of the sample period. Traffic is well-known as an emission source of particle number concentration especially of ultrafine particles [39]. In our study the ultrafine particles concentrations were 10344 and 10802 1/cm^3 ^for the mean of the sample period. The applied sample and resuspension technique (reference method of US-EPA) provided PM and the water soluble organic and inorganic constituents from ambient Augsburg PM to the rat lungs by instillation. Although the sampled PM_2.5 _includes the ultrafine particles fraction it is unlikely that sampling and preparation keeps ultrafine particles in its "airborne state" rather ultrafine particles may form aggregates with itself or fine particles but they may keep their surface toxicity.

### Cardiovascular performance

As expected, cardiovascular effects in SHRs at high levels of PM_2.5_-AB were small and would, as a single event be clinically not relevant among subjects with adequate cardiovascular reserves, or even not in patients with cardiac diseases. However, triggering effects by repeated exposures over several years as given in the environmental setting of Augsburg may be one major factor contributing to the promotion of cardiovascular disorders as suggested by the epidemiological associations. The response in SHRs at high levels of PM_2.5_-AB was characterized by a 5% increase in mBP in exposed animals between the 2^nd ^- 4^th ^post exposure days the maximum being on 4^th ^day after instillation. Changes in mBP were due to a moderate changes in systolic and diastolic BP. HR decreased by about 6% on the 3^rd ^day after instillation. However, the statistical analyses were not definitely decisive for these cardiovascular effects so that they have to be interpreted with caution and the results valued in context with the effects detected for markers of vascular homeostasis. In other experimental settings moderate HR and BP changes have repeatedly been reported in response to different PM [44-47] such as an increased HR and BP in SHRs exposed to ultrafine carbon particles [45] but also a decreased HR in response to highway aerosols [44]. The direction and time point of HR and BP differed between studies as the PM composition and fraction used suggesting that the cardiovascular response to ambient particle may result from a summation of temporally different effects of PM-associated components including direct interaction of translocated particles to pulmonary and systemic tissues [48,49]. The BP changes observed in the present study are in line with the epidemiological findings revealing a slight increased arterial BP in response to elevated levels of Augsburg PM [25], in particular in compromised subjects with high plasma viscosity levels. However, changes in epidemiological studies were detected at an earlier time point than in our experimental setting and HR rather increased than decreased [9]. Different factors one may speculate on may account for this, such as randomly selected population versus rats with distinct cardiovascular disease, species difference, route of PM exposure and particle translocation kinetics, but at hand it is beyond the scope of the present study to provide an obvious explanation.

### Pulmonary and systemic inflammatory responses

A single Intratracheal instillation of PM_2.5_-AB was associated with a low grade dose-dependent pulmonary inflammation as indicated by accumulation of particle-laden macrophages, lymphocytes, and PMNs resulting in a 2-fold induction of total cell numbers in BALF on the 1^st ^day post instillation. This cellular response was resolved within 3 days. In contrast, PM mediated in vivo toxicity screening studies in SRHs by Gerlofs-Nijland et al [36] reported a tremendous increase in PMN number in BALF 24-hr after exposure to the highest concentrations (10 mg/kg bw i.t.) of EHC-93 (urban dust from Ottawa, Canada) or PM_10 _sampled nearby a road tunnel. This suggests that PM from Augsburg has less inflammatory potential and that the severity of health effects induced by PM from different sampling sites differs substantially between sites most likely due to differences in chemical composition and the relative share of the particle size fractions at the different sites.

The dose-dependent induction of the proinflammatory cytokine (IL-6) and the matricellular (matrix+cytokine: osteopontin; [50]) protein in BALF confirm the PM_2.5_-AB mediated pulmonary inflammation. Corresponding observations were made in the epidemiological studies in Augsburg [34,51] and have also been reported in other studies [30,52].

Panel and population studies have associated elevated PM levels with evidence of an acute-phase response inferred from increased serum CRP levels [20], enhanced plasma viscosity [17] and altered hematologic indices [32,33,53,54]. Epidemiological findings [34,51] have suggested that the increased level of IL-6 following high ambient PM load possible leads to the production of acute phase proteins like CRP. This fits to observation made in the present study where peak IL-6 concentrations in the BALF were observed on the 1st day after instillation of PM_2.5_-AB and highest CRP concentrations in serum after the 3rd day. In a recent study Zhao et al. [55] have reported that the levels of CRP in serum were significantly higher in SHRs than those in WKY following instillation of fine particle (8 and 40 mg/kg; collected from Shanghai, China) supporting the notion that individuals with persisting cardiopulmonary disorder are at higher risk when exposed to the elevated level of PM than healthy ones.

Serum CRP concentrations reflect the burden of vascular inflammation. In the presence of an intercurrent illness or an exogenous inflammatory stimulus, serum CRP concentrations increase rapidly as part of the systemic acute-phase reaction [56,57]. In recent years CRP has also come up as a leading biological marker of atherosclerosis, with serum CRP concentrations, even at very low levels, predicting the risk of acute myocardial infarction or stroke in apparently healthy individuals [34,57]. CRP seems to be a strong predictor of cardiovascular events in humans. Using widely available high-sensitivity assays, CRP levels of <1, 1 to 3, and >3 mg/L correspond to low-, moderate-, and high-risk groups for future cardiovascular events in human [58]. We are not aware of such grading for rodents. But if we extrapolate from the available information in humans, a ~4 fold induction of serum CRP on the 3rd day post instillation of 1000 μg PM_2.5_-AB suggests high risk for cardiovascular impairment in SHRs.

The time and dose dependent induction of MIP-2 and osteopontin in cardiac tissue suggests that the PM_2.5_-AB mediated sustained systemic inflammatory response also involves cardiac tissue. Although cellular mechanism of systemic inflammation are very complex, a number of researchers reported that the induction of chemokine (MIP-2) and the upregulation of matricellular protein (ostopontin) are involved in the recruitment and stimulation of neutrophil, macrophage, and lymphoctes after particle exposure [59].

The present findings are consistent with a series of studies suggesting that intratracheal instillation of PM in rodents caused pulmonary and systemic inflammation [60-63]. Effect of pulmonary PM exposure by inhalation may be with/without systemic inflammatory response [45]. This is mainly due to the lower dosage delivered to the lungs over a longer period but the observed effect also depend on the chemical composition, presence of water-soluble metals and surface area of the particles as shown in various studies [62-64].

### Responses affecting vascular homeostasis

Potential pathways linking the observed pulmonary and systemic events are identified by analysis of various biomarkers associated with hypertension (ACE), endothelial activation (ET-1), coagulation factors (TF; PAI-1), atherosclerosis (VACM-1) and angiogenesis (VEGF) from pulmonary and cardiac tissues. Protein analysis of these markers in cardiac tissues revealed a delayed systemic response while the same markers responded differently or to a lesser extent in pulmonary tissues. This result illustrates the PM_2.5_-AB-specific differences in the degree and time course of pulmonary and cardiac effects. Studies from Wallenborn et al. [64] support our observation. They specifically addressed the differential pulmonary and cardiac effects of intratracheal instilled metals in WKY rats (1 micromol/kg body weight of zinc, nickel, vanadium, copper, or iron in sulfate form). Depending on the metal species applied, the severity and the time point of pulmonary inflammation varied. Similarly, the systemic effects noted for zinc, nickel or copper occurred at different time points.

In pulmonary tissue a transient and early induction of PAI-1, VEGF, and VCAM-1 and a decline in ACE, ET-1, and TF concentrations suggests direct involvement of the pulmonary vasculature in the PM_2.5_-AB response. Reduced ACE and ET-1 levels may indicate a vasodilation response as similar to the other studies [65,66]. Increased expression of PAI-1 in the vasculature has been recognized as indicator of disturbed endothelial function [67] and associated with the progression of atherosclerotic lesions [68,69]. Many studies have shown that alteration of the VEGF homeostasis is involved in different pathological states of pulmonary tissues, such as acute lung injury and pulmonary arterial hypertension [70,71]. A moderate upregulation of VEGF - as observed in our study - is often regarded as a protective mechanism to restore tissue homeostasis because VEGF serves as a survival and differentiation factor for pulmonary endothelial cells [70,72]. Up-regulation of VCAM-1 in the pulmonary tissue might be due to the activation of endothelial cell by cytokines released from activated macrophages or recruited PMNs [70]. Different studies [73-75] have provided evidence that VCAM-1 promotes the progression of atherosclerosis by accumulation, adhesion and trans-endothelial migration of leukocytes. Furthermore, activated endothelial cells can increase the expression of PAI-1, VEGF and VACM-1 [70,76] and might be responsible for development of atherosclerosis lesion.

Interestingly, in the heart all measured markers (except VEGF) were increased at high concentrations on day 3 post exposure. The observed increased levels of PAI-1, known to be an inhibitor of fibrinolysis and regulator of vasoactivity, have been recognized as indication of impaired endothelial function [67]. The additionally observed induction of TF, the extrinsic coagulation pathway, is highly correlated with thrombogenesis and endothelial dysfunction [77,78]. Therefore activation of TF in association with impaired fibrinolysis via PAI-1 activation following PM_2.5_-AB exposure supports the notion of endothelial dysfunction, which is importantly correlated with overall cardiovascular risk [77,79]. Endothelial dysfunction by PM in cardiac tissue can be further inferred from induction of ET-1 and activation of the ACE system in cardiac tissue following instillation of high PM_2.5_-AB. Interestingly, the slight increase in BP follows the kinetics of ACE and ET-1 concentrations in serum. ACE and other components of the RAS (including rennin and angiotensin II receptor) are expressed in the several organs (e.g. lung, heart, brain) and play a significant role in the regulation of systemic BP and endothelial homeostasis.. Several studies reported that RAS system plays a key role in ET-1 expression and might also be responsible for up-regulation of ETB receptors [80,81]. Overall, PM_2.5_-AB exposure at high concentration affects different endpoints of the vascular homeostasis in the lungs and the systemic circulation by triggering pathways known to be involved in endothelial dysfunction, vasoregulation, and thrombogenesis.

In this study physiological parameters like HR and HRV responded differently compared to our previous ultrafine carbon particle inhalation study in SHRs [45]. Additionally we detected alteration at molecular level to be more pronounced in cardiac tissue compared to pulmonary tissue previously. This may be related to the different age groups (12 months versus 6 months) of animals investigated and plausibly because of the altered organ functions due to hypertension. But it's more likely that beside carbon particles other ambient PM2.5 components from Augsburg are involved in the observed systemic response [82]. Chemical analysis of PM from Augsburg urban area reported the presence of anthropogenic polycyclic aromatic hydrocarbons (PAH) and their oxidised derivatives (O-PAH, 40-43). Concentrations of PAH and O-PAH in PM were highly correlated to ROS formation, which is known to be involved in inflammatory responses in the lungs and extrapulmonary organs [83]. Beside that PM_2.5_-AB contains various transition metals which may move as soluble components directly through the pulmonary vasculature and reach the systemic circulation where the metals exert direct effects on extrapulmonary tissue [62,64].

## Conclusion

Although there are some differences between the effects observed in our animal study and the effects detected by epidemiological studies in Augsburg, overall, there is clear evidence that many different pathways are triggered in our model which play an important role in cardiovascular impairment and fatal events. A schematic summarization of the results obtained from Augsburg dust exposures in SHRs in different organs are presented in Figure 8. The scheme mechanistically links the observations obtained in this study as potential underlying factors for the fatal cardiovascular events recognized in epidemiological studies. As observed in many experimental studies PM_2.5_-AB instillation induced a transient inflammatory response in the lungs which was largely resolved within 3 days. VEGF was moderately increased presumably involved in restoration of lung tissue homeostasis, whereas continued effects are still observed in the pulmonary vasculature. They were associated with coagulation activity and vasoregulatory pathways including the renin-angiotensin system. Secondary systemic effects were characterized by a delayed, low grade systemic inflammatory response not only involving the vascular compartment (CRP) but also in cardiac tissue (Osteopontion, MIP-2). Through the induction of the coagulation cascade (PAI-1, TF, VCAM-1) and vasoregulatory activities (ACE, ET-1) PM_2.5_-AB transiently augments the hypertension and may contribute to the development and progression of atherosclerotic lesions. These acute effects underpin the atherothrombotic consequences of acute exposure to PM2.5-AB on cardiovascular disease. Although the chronic exposure as missing link is lacking and beyond the scope of the present study it is reasonable to consider effects of a single exposure to be small and, taken as an unique event, to be clinically of minor importance. However, repeated exposures over several years as given in the environmental setting of Augsburg is supposed to add up over time [84] and promote cardiovascular disorders in susceptible-predisposed individuals, which may eventually contribute to trigger fatal cardiac events such as myocardial infarction documented in the epidemiological studies (Figure 8).

## Materials and methods

### Sampling Location

The sample site was placed on the campus of the University of Applied Sciences Augsburg which is approximately 1 km to the south-east of the city centre. Within a radius of 100 m it is surrounded by campus buildings, a tram depot and a small company. The nearest main road is in the north-east at a distance of 100 m. Within a radius of approximately 200 m the monitoring site is almost completely surrounded by residential areas, apart from a small park located in north-western direction.

### PM sampling

We used a High Volume Sampler (HVS, model DHA-80, DIGITEL Elektronik AG, Switzerland) equipped with a PM_2.5 _pre-separator (one-stage impactor certificated according to EN12341) to collect PM_2.5 _samples on filters (diameter 150mm). The flow rate of the HVS was adjusted to 30 m^3^/h and automatic regulated. After sampling the filters were frozen at -20°C for further resuspension.

The sampling period started on the 18^th ^of September 2006 and ended on the 16^th ^of October 2006. In total 8 filters were available for an alternating sampling period of 3 and 4 days.

### Ambient concentrations (mass concentration of PM_2.5_, number concentrations of ultrafine particles)

The mass concentration of PM_2.5_-AB was measured using a tapered element oscillating microbalance (TEOM, model 1400ab, Thermo Fisher Scientific Inc., U.S.) equipped with a PM_2.5 _inlet. To correct the PM measurement for aerosol volatility effects, the TEOM was equipped with a filter dynamics measurement system (FDMS, model 8500b, Thermo Fisher Scientific Inc., U.S.).

The UFP number concentration was calculated from the particle size distribution measured with a twin differential mobility particle sizer (TDMPS) system. The TDMPS system consists of two subsystems covering different size ranges.

Details on the used online measurement devices and further information about other continuously measured physical and chemical parameters were already published by Pitz et al. [38,39]

#### Animals

Male spontaneously hypertensive rats (SHR) were used for the present study. Animals were housed under filtered air and specific pathogen free (SPF) conditions at a mean temperature of 22 ± 2°C, a mean relative humidity of 50 ± 5%, and a 12 h light-dark cycle (6 a.m. to 6 p.m. light on) with pelleted feed and filtered water being supplied *ad libitum*. Experimental protocols were approved by the Animal Care and Use Committee of the HelmholtzZentrum München - German Research Center for Environmental Health and by the Bavarian Animal Research Authority (211-2531-88/2007). Animals were 12-months of age with body weights between 360-380 g during the study.

#### Intra-tracheal instillation study

To assess the inflammatory response following instillation of ambient particle two animal groups were used. In this study 1 year old male SHRs of Group A (Sham/exposed: n = 8/8; Table 2) were IT-instilled with either pyrogene-free distilled water (sham) or water suspension of ambient particle (PM_2.5_-AB) at 250 μg, 500 μg and 1000 μg under isoflurane anaesthesia and lavage were performed at 24 h or day 1 postinstillation. Similarly Group B (Sham/exposed: n = 8/8; Table 2) animals were IT-instilled either pyrogene-free distilled water (sham) or water suspension of ambient particle (PM_2.5_-AB) at 500 μg and 1000 μg under isoflurane anaesthesia and lavage were performed at 72 h or day 3 postinstillation. Additionally we have performed cardiovascular response study following instillation of pyrogene-free distilled water (sham; n = 5) and 1000 μg of particle (exposed; n = 5), as inflammatory response were more pronounced at 1000 μg. For intra-tracheal instillation SHRs were anesthetized by administration of isofluran (5.0% isoflurane in O2; Sigma Delta Vaporizer; Uno, Zevenaar, Netherlands) using a customized flow-through mask (Helmholtz-Zentrum München, Neuherberg, Germany). The animals were then intubated by a nonsurgical technique [85] using a bulb headed cannula inserted 10 mm into the trachea; 250 μl of a suspension containing 250, 500 and 1000 μg particles, respectively, in pyrogene-free distilled water was instilled individually in each animal. The suspension of particles was sonicated for 1 min prior to each instillation, using an ultrasonic bath SonoPlus HD70 (Bachofer, Berlin, Germany) at a moderate energy of 20 W. We favour the use of pyrogene-free distilled water for suspension of particles because the salt content of phosphate-buffered saline (PBS) causes rapid particle agglomeration comparable to the "salting-out" effect [63] and thus eliminates consistent instillation conditions. Control (sham) animals were instilled with pyrogene-free distilled water.

**Table 2:** Experimental design of cardio physiological, pulmonary and systemic response..

Instillation								
	**Adaptatiion**		**Baseline**	**Post exposure**				

**Cardiovascular response**	**Day -2**	**Day-1**	**Day 0**	**Night 1**	**Night 2**	**Night 3**	**Night 4**	**Night 5**

	Acclimatization		√	√	√	√	√	√

Telemetry (n = 5)*	No change		BP	BP	BP	BP	BP	BP

			HR	HR	HR	HR	HR	HR

***ambient particle-mediated pulmonary and systemic response***								

**Pulmonary resposne**	**Day 1 (24 h; Group A)**					**Day 3 (72 h; Group B)**		

**Dose/rat**	250 μg	500 μg		1000 μg		500 μg	1000 μg	

BALF (n = 6)	√	√		√		√	√	

Lung tissue (n = 6)	√	√		√		√	√	

Pulmonary histopathology (n = 2)	√	√		√		√	√	

**Systemic response**								

Blood (n = 8)	√	√		√		√	√	

Cardiac tissue (n = 6)	√	√		√		√	√	

Cardiac histopathology (n = 2)	√	√		√		√	√	

*Number of exposed animals is given. A similar number of animals were intratrachealy instilled with pyrogene-free distilled water and served as controls or sham. **Blood **: blood collected from retro arbitral sinus and abdominal aorta.. **BP **and **HR**: blood pressure and heart rate were the two primary parameters measured for the cardiovascular response.

#### Assessment of ambient particle-mediated pulmonary inflammatory response

##### BALF and lung

BALF analysis was performed on day 1 (24h: group A) and day 3 (72h: group B) after instillation as described in our previous study [45,86]. In brief, one aliquot of whole BALF (n = 6) was used for determining total cell counts (Coulter Counter; Coulter, Inc., Miami, FL), and a second aliquot was centrifuged (Cytospin 2; Shandon, Astmoor, UK) to counts cell differential. Macrophages, polymorphonuclear cells (PMNs, or neutrophil), eosinophil, and lymphocyte were counted using light microscopy (over 200 cells counted per slide). The remaining BALF was centrifuged (1500 × g) to remove cells, and the supernatant fluids were analyzed for osteopontin (Catalog # 900-089; Biomol) and IL-6 (Catalog # 550319; BD-Bioscience) concentration, as potential biological markers for pulmonary inflammation. Furthermore, transcript profiling markers associated with pulmonary inflammation (MIP-2, TNF-α), were also assessed at protein level from the pulmonary tissues (n = 6) using the Rodent MAP™ version 2.0-custom 8-plex of the Rules Based Medicine (Austin, Texas).

##### Pulmonary histopathology

The left lung of each non lavaged animal (n = 2) was infused via left main bronchus by 4% buffered formalin at 20 cm water pressure for 20-30 minutes. The main bronchus was then tied and the lung was submerged in fixative until processing for histology. Paraffin blocks were prepared from dehydrated tissues and 3- to 4- μm sections were stained with hematoxylin and eosin for light microscopic evaluation of the pulmonary tissues [45,86]..

#### Assessment of ambient particle-mediated effects on pulmonary and cardiac tissue

##### Protein analysis

For protein analysis lung and cardiac tissues were collected from each animals (n = 6) immediately after BALF collection, placed in vials and flash frozen in liquid nitrogen. They were then stored at -80°C until lung and heart tissue homogenates were prepared.

##### Pulmonary and Cardiac tissue homogenate preparation

Total lung and heart homogenate was prepared using 50 mM Tris-HCL with 2 mM EDTA, pH 7.4 as the lysis buffer (1000 μl) from 6 animals/experimental group. Using the Rodent MAP™ version 2.0-custom 8-plex of the Rules Based Medicine (Austin, Texas) a panel of inflammatory and biomarkers (protein) associated with hypertension (ACE), endothelial activation (ET-1), coagulation factors (TF; PAI-1), atherosclerosis (VACM-1) and angiogenesis (VEGF) were analyzed from the lung and heart homogenate. Lung and heart homogenate of the n = 6/6 (sham/exposed) were used for the measurment and only the markers equal to/above (≥) the sensitivity level were considered. Sensitivity level is the least detectable dose (LDD) as provided by Rules Based Medicine. In all cases a strong homogeneity of data were observed. Osteopontin (SPP1) was also assayed from the heart homogenate samples by ELISA (Catalog # 900089; Biomol).

##### Cardiac histopathology

The whole heart of each non lavaged animal (n = 2) was submerged in fixative until processing for histology. Paraffin blocks were prepared from dehydrated tissues and 3- to 4- μm sections were stained with hematoxylin and eosin for light microscopic evaluation of the cardiac histopathology [86] .

#### Assessment of ambient particle-mediated systemic response

Haematological analysis, measurement of different biomarkers from plasma and serum were used for the assessment of systemic response following PM_2.5_-AB exposure. Blood samples of each animal were collected from retro orbital sinus (haematology) and from abdominal aorta (biomarkers) on day 1 and day 3 after instillation.

##### Haematology

For haematological analysis, 500 μl of blood sample from retro orbital sinus of each animal (n = 8) was collected in EDTA-Microvette and analysed by using haematology analyzer (Bayer ADVIA 120, Germany).

##### Acute phase proteins analysis

Blood samples collected from each animal (n = 8) were stored in aliquots of 2.6 ml in 2.9 ml *S-Monovette*^® ^tube (Sarstedt, Germany) without anticoagulant for further analysis of biomarker. C-reactive protein (CRP) was analysed from serum collected from blood samples after centrifugation for 15-minutes (at 1300g, 4°C). CRP was measured by ELISA (Catalog#: 557825, Biomol). Furthermore, the markers associated with systemic inflammation (MIP-2, TNF-α), were also assessed at protein level from the cardiac tissues (n = 6) using the Rodent MAP™ version 2.0-custom 8-plex of the Rules Based Medicine (Austin, Texas).

### Cardiophysiological analysis by radiotelemetry

#### Exposure protocol

Cardiophysiological response prior to and following instillation of ambient particle (1000 μg/rats) was performed on 12 months old SHRs by using radio telemetric system as described in our previous study (86, Dataquest A.R.T; Data Sciences International D.S.I., St. Paul MN, U.S.A). The implantation of telemetric devices into the peritoneal cavity of animals was performed as previously described [86]. All animals exhibited rapid post surgical recovery, with resumption of normal food and water intake within 24 h of surgery. They returned to presurgical body weight (excluding the weight of the implant) on average within 3-4 days and did not exhibit any signs of post surgical complications. After 10 days of post surgical recovery, 10 animals were randomly assigned to the control (sham) or the exposure group, respectively. Data recording was then initiated and continued for six days, that included a baseline reading (day 0), and post exposure period readings (days 1-5) after instillation of pyrogene-free distilled water (sham) or 1000 μg ambient particle (exposed, Figure 2). In this study separate animal groups (n = 5) were used for sham (instilled with water) and exposed (instilled with 1000 μg particle) SHRs.

#### Animal preparation, data acquisition and analysis using radio telemetry system

The implantation of telemetric devices into the peritoneal cavity of animals (sham/exposed: n = 5/5), the radio telemetric data acquisition and analysis were performed as described previously [45,86]. Briefly, arterial BP, HR, body core temperature (T), and physical activity (*Act*) of SHRs were continuously collected over 24h/day, throughout baseline and after instillation for 5 days. Systolic (sBP), diastolic (dBP), and mean (mBP) arterial blood pressure were determined from the BP tracings on a beat to beat basis. The data of each animal were then processed to obtain 10-minutes average segments per rat for each of the measured parameters. For the final data analysis, we only have considered the values of each parameter from the 12 h dark period (6 p.m. to 6 a.m.), as animals are more active during the night time. Thereby, 72 consecutive values of 10-minutes data segments were obtained per rat per day and per parameter. Since we did not observe a time dependency of particle associated effects during the 12 h period in each of the exposed animals, mean values were used for further data processing. For all of the measured parameters, we averaged the 72 values obtained for each day resulting in one mean value per parameter per rat and per day. Based on these values, group mean were calculated on a daily bases for the whole study and were used for statistical comparison between sham (pyrogene-free distilled water) and exposed (1000 μg particle) SHRs.

For heart-rate variability (HRV) analysis, a different procedure has to be applied [86]. For the 12 h dark periods, one 5-minutes ECG segment per hour was randomly selected and used for further HRV analysis. For each of these 5-minutes segments the standard deviation of all adjacent normal sinus NN intervals (SDNN) was determined as a measure of the overall HRV. In addition, the square root of the mean of squared differences between adjacent normal to normal intervals (RMSSD) and the low-frequency to high-frequency ratio (LF/HF), reflecting the balance of cardiac parasympathetic tone and sympathetic activity, respectively, were determined. Further data processing to obtain daily averages for each of the rats and group averages followed the procedure described above for the other parameters.

### Statistics

After checking for the normal distribution assumption the differences between exposure and control (sham) groups were compared by using the t-test. Cardiovascular response parameters were described by a linear mixed regression model for repeated measurements. Based on this model group differences between the exposure and control group were tested. To assess whether systematic BP and hear rate changes occur after PM_2.5 _instillation area under the curve (AUC) statistics was applied as an summary indicator of systematic changes for repeated measurements. For protein analysis of various parameters from lung and heart tissues, a two-way analysis of variance (ANOVA) was used to analyze differences between the groups. P values less than 0.05 were stated as statistically significant. All computations were done by the software packages Statgraphics plus v5.0 (Manugistics, Rockville, MD) and SAS V9.1 (Cary, NC). Data are presented as arithmetic mean values of n observations ± the standard error (SE), unless otherwise indicated.

## Competing interests

The authors declare that they have no competing interests.

## Authors' contributions

SU KG, TS, MP, PR, WGK, AP, HEW, HS conceived and designed the experiments. SU, KG, TS, ST, MSB, MP performed the experiment; SU, KG, TS, ST, PR, MP, HS analyzed the data; SU, KG, TS, MP, WGK, OE, HEW, HS wrote the manuscript.

All authors read and approved the final manuscript.

## About the authors

From the Comprehensive Pneumology Center, Institute of Lung Biology and Disease (SU, KG, ST, MSB, TS, WGK, OE, HS); Focus Network: Nanoparticles and Health (WGK, HEW, HS), Institute of Epidemiology (MP, AP, HEW), Institute of Health Economics and Health Care Management (PR),. Helmholtz Zentrum Munich: German Research Center for Environmental Health.
